# Evaluation of methods for AlphaFold-based integrative modeling

**DOI:** 10.64898/2026.09.13.751323

**Published:** 2026-09-16

**Authors:** Kartik Majila, Shruthi Viswanath

**Affiliations:** 1 National Centre for Biological Sciences, Tata Institute of Fundamental Research, Bangalore, Karnataka, India 560065.

**Keywords:** AlphaFold, Integrative modeling, Macromolecular assemblies, Structure prediction, Chemical crosslinking

## Abstract

**Motivation:**

Recent methods enable incorporation of experimental data into AlphaFold for predicting structures consistent with the data. However, the applicability of these methods for integrative modeling remains to be determined. It is unclear how these methods balance the input experimental data with the learned structural priors.

**Results:**

We assess the performance of state-of-the-art AlphaFold-based integrative modeling methods, including AlphaLink2, Boltz2, and GRASP, on a dataset of 37 complexes based on crosslinking data. We evaluate these methods based on their ability to predict structures that satisfy the input crosslinks. We further assess the robustness of these methods to noise in the crosslinking data. Finally, we probe their ability to predict distinct states using crosslinks from multiple states. Overall, our study highlights the limitations of the AF-based IM methods and points to directions for future improvements.

**Availability and implementation:**

All scripts used to obtain the predictions and perform the analysis in this study are available at https://github.com/isblab/af_im.

## Introduction

Macromolecular complexes play important roles in various cellular processes, including transcription, translation, molecular trafficking, and signal transduction, yet characterizing their three-dimensional structures remains challenging ^1,2^. Often, experimental structure determination techniques like X-ray crystallography, nuclear magnetic resonance (NMR), and cryo-electron microscopy (cryo-EM) remain limited due to challenges in sample preparation and the size and heterogeneity of the complexes ^1,3^. Integrative modeling (IM) is an approach that overcomes the limitations of individual techniques by integrating data from various experimental techniques along with physical principles, statistical inferences, and prior models ^1,4–7^. Given sparse, noisy, and ambiguous input data from heterogeneous sources, no single structure may satisfy all the data. As a result, the aim of integrative modeling is to determine the set of all structures consistent with the input data ^1,3,7^.

Advancements in deep learning methods like AlphaFold (AF) have transformed structural biology, enabling accurate sequence-based structure prediction ^8–10^. Although methods such as AlphaFold-multimer and AlphaFold3 enable modeling complexes, they are often inaccurate on challenging targets, including large complexes, disordered proteins, and multi-state proteins ^11–14^. Recent methodological advances have led to the development of AlphaFold-based integrative modeling (AF-based IM) methods that allow data-guided structure prediction ^13^. Examples include AlphaLink/AlphaLink2, GRASP, Distance-AF, ColabDock, ROCKET, Guided-AF3, ConforNets, and Boltz2 ^13,15–22^. In contrast to methods that use AlphaFold predictions as input for downstream modeling, AF-based IM methods allow incorporating experimental data to influence the AlphaFold predictions ^13^. The experimental data is incorporated in various ways: as a bias for the MSA and pair representations, as restraints in the loss function for fine-tuning, or as a bias in the diffusion sampling process ^13^. Specifically, AlphaLink/AlphaLink2 incorporates crosslinking data into the pair representation used as input to AlphaFold’s Evoformer ^19,20^. Similarly, GRASP incorporates residue-level interaction data into the MSA representation input to the Evoformer and the single representation input to the structure module ^21^. In contrast, Boltz2 incorporates distance restraints from experimental data to bias the diffusion sampling at inference time. Thus, by combining AlphaFold’s learned structural priors with biophysical information, these methods potentially enable data-guided conformational search ^18^.

Despite the rapid progress of these AF-based IM methods, their applicability and limitations have not been systematically evaluated. It remains unknown how these methods weigh the learned structural priors with experimental restraints, particularly when dealing with noisy data and data derived from multiple states. In this study, we benchmark the performance of state-of-the-art AF-based IM methods – AlphaLink2, Boltz2, and GRASP – on a dataset of 37 multi-protein complexes using chemical crosslinks as input. We assess the ability of these methods to predict structures consistent with the input crosslinks. We evaluate the set of predicted structures from these methods based on their accuracy, stereochemical plausibility, and diversity. We also test the robustness of these methods to noise in the crosslinking data. Finally, we probe their ability to predict distinct states based on crosslinks from multiple states. Overall, our study highlights several limitations of the AF-based IM methods and points to directions for future improvements.

## Results

**Figure 1:**
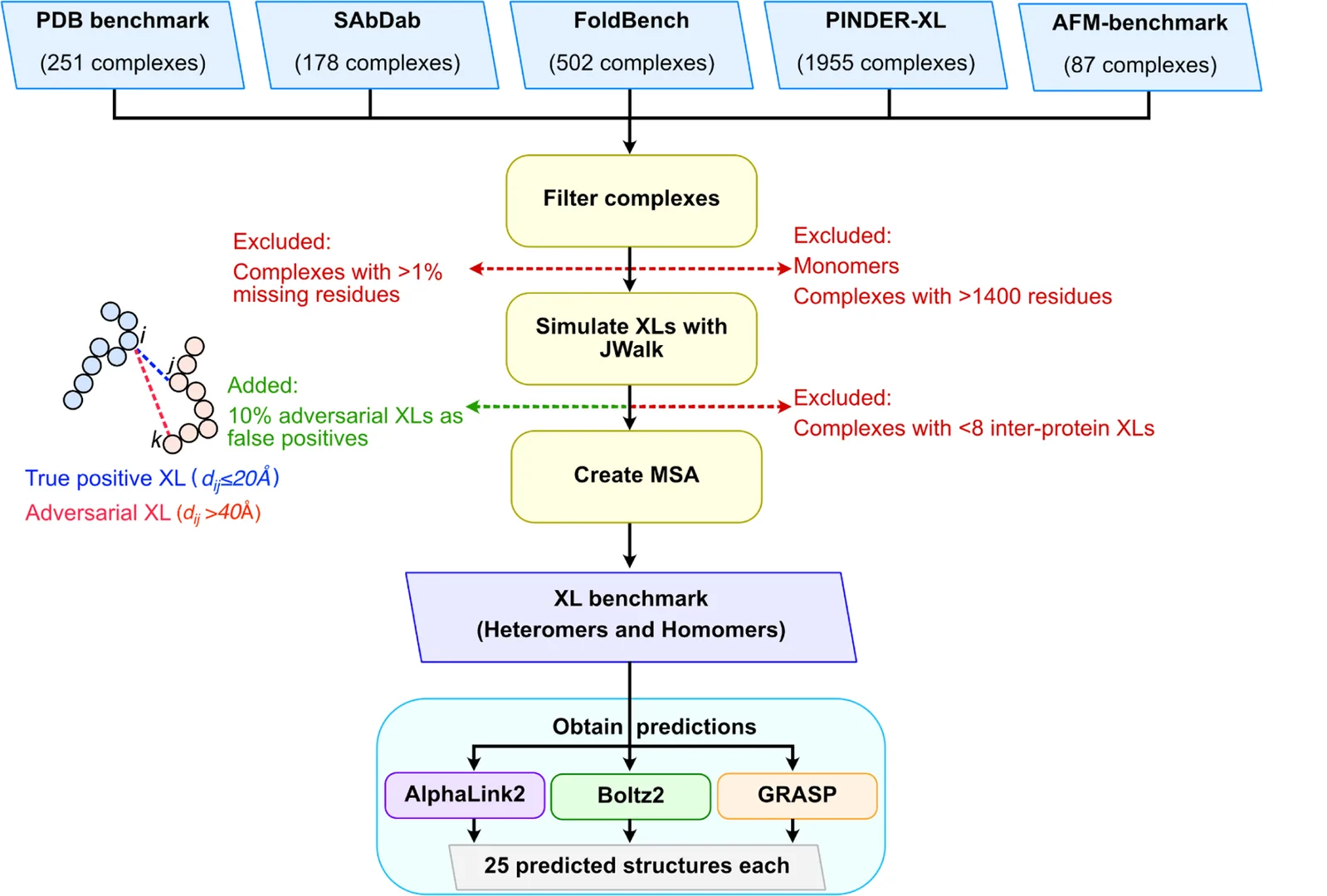
Dataset creation pipeline. Our dataset was compiled by gathering protein complexes from existing benchmarks, including the PDB benchmark, SAbDab, FoldBench, PINDER-XL, and AFM-benchmark. Monomeric proteins, complexes with missing residues, and those greater than 1400 residues in length were excluded. Inter-protein crosslinks (XLs) for each complex were computed using JWalk, and complexes with fewer than eight crosslinks were excluded. Residue pairs with a solvent accessible surface distance (SASD) less than 20 Å were considered as true positive (TP) crosslinks. Additionally, 10% adversarial false positive (FP) crosslinks with a SASD greater than 40 Å were used. MSAs for each complex were computed using the OpenFold MSA creation pipeline. Three AF-based IM methods were selected – AlphaLink2, Boltz2, and GRASP for evaluation.

To assess the performance of the AF-based integrative modeling methods, we created a benchmark of 37 complexes with simulated crosslinks obtained from JWalk (Fig. 1, Suppl Table 1, see Methods). Input crosslinks were simulated from the known experimental structure (native structure) of each complex. Ninety percent of these crosslinks were consistent with the native structure (SASD≤20 Å; true positives), whereas the remaining 10% were adversarial crosslinks inconsistent with the native structure (SASD>40 Å; false positives), reflecting the false positive rate observed in crosslinking experiments (Fig. 1). Three AF-based IM methods were evaluated: AlphaLink, Boltz, and GRASP, with twenty-five structures predicted by each method per complex.

### Do the predicted structures satisfy the data?

**Figure 2:**
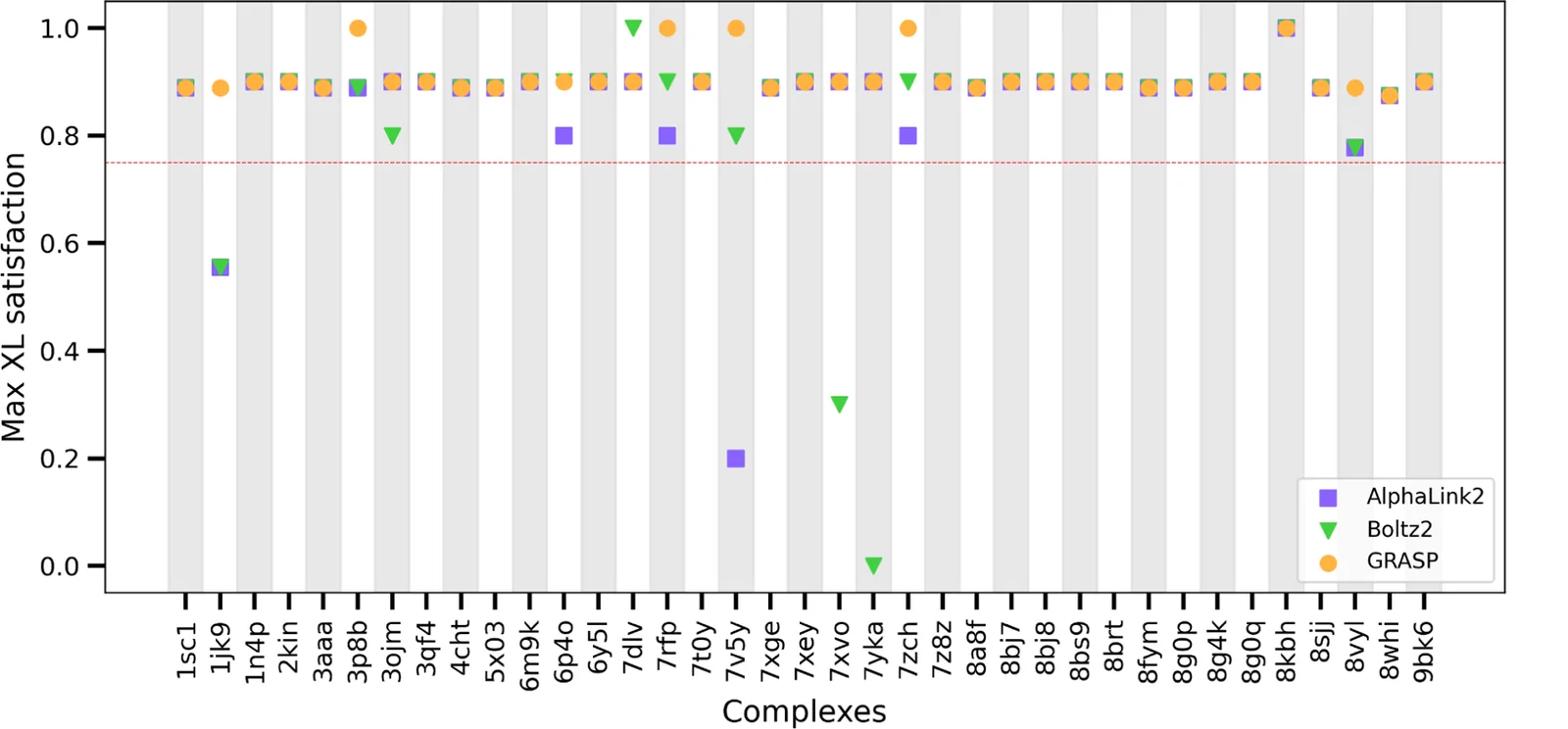
Assessing crosslink satisfaction. For each complex, the maximum crosslink (XL) satisfaction achieved by a structure in the set of predicted structures is shown for AlphaLink2 (purple, squares), Boltz2 (green, triangles), and GRASP (orange, circles).

First, we assessed whether the AF-based IM methods could predict structures that satisfied the input crosslinks. The crosslink satisfaction for a predicted structure is the fraction of crosslinks satisfied by the structure. We measured the maximum crosslink satisfaction achieved across all predicted structures from each method for each complex. For most complexes, all the methods predicted at least one structure with high crosslink satisfaction, *i.e.,* more than 75% crosslinks satisfied (Fig. 2). GRASP predicted structures with high crosslink satisfaction for all the complexes, whereas AlphaLink2 and Boltz2 predicted structures with high crosslink satisfaction for 35 and 34 complexes, respectively (Fig. 2, Supp Table 1).

### Do guided predictions improve data satisfaction?

Having established that the AF-based IM methods could predict structures consistent with the input crosslinks, we next examined whether using crosslinks to guide the prediction improves crosslink satisfaction relative to unguided predictions, in which no crosslinks are provided. For this, we compared Boltz2 and GRASP, since they allowed performing unguided predictions, whereas AlphaLink2 does not. Comparing the distribution of per-model crosslink satisfaction between guided and unguided predictions, we observed that crosslink guidance improves crosslink satisfaction for GRASP (Supp Fig. 1, 2A). However, for Boltz2, the per-model distributions were nearly identical for both guided and unguided predictions, potentially suggesting a strong training bias (Supp Fig. 1, 2B).

### Are the predicted structures close to the native state?

**Figure 3:**
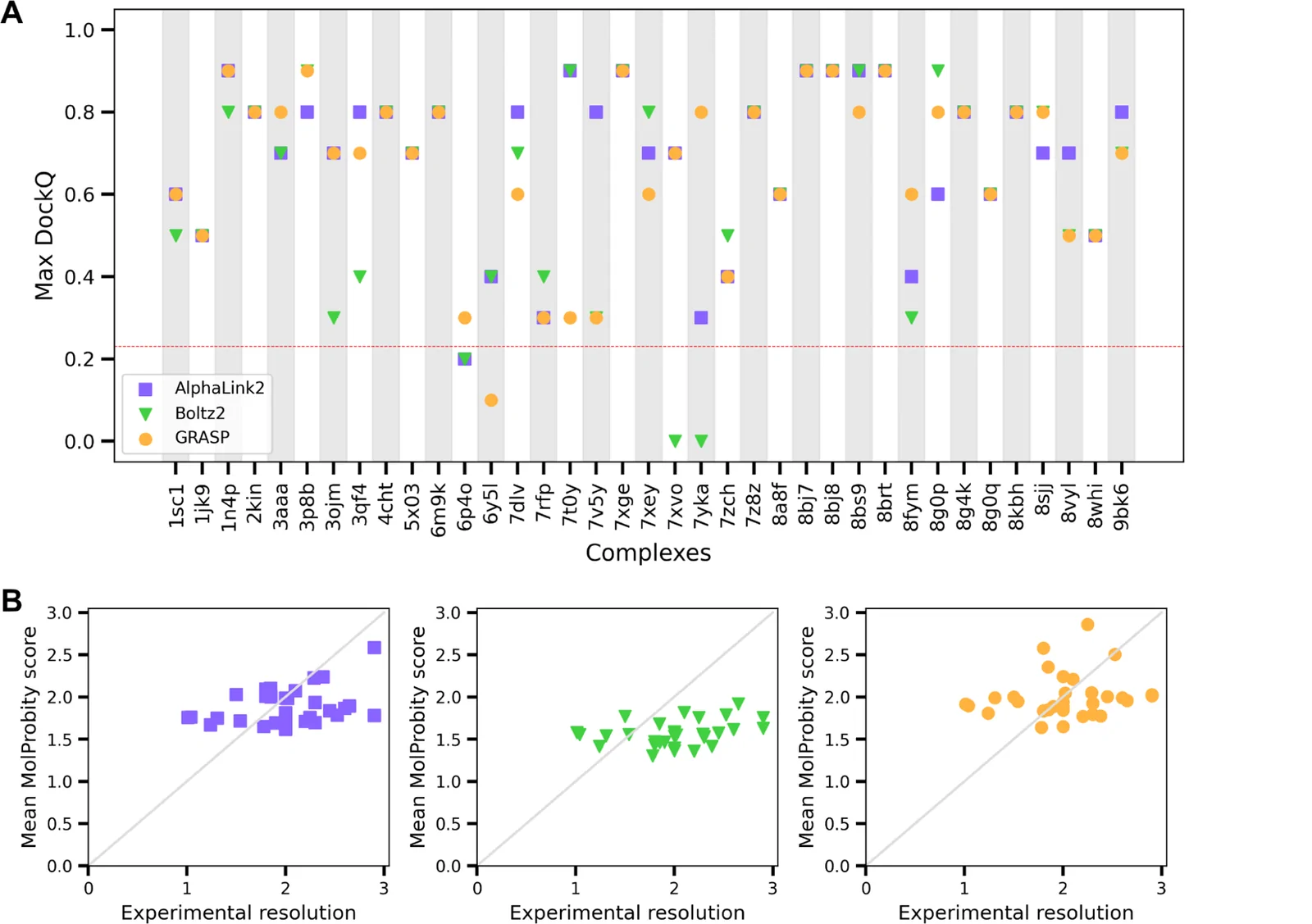
Assessing structural accuracy and stereochemical plausibility. For predictions obtained from AlphaLink2 (purple, squares), Boltz2 (green, triangles), and GRASP (orange, circles), (A) the maximum DockQ score achieved across all predicted structures for each complex is shown as a measure of structural accuracy, and (B) the mean MolProbity score across all predicted structures is shown as a function of the experimental resolution of the native structure.

High crosslink satisfaction alone does not imply structural accuracy, *i.e.,* similarity of the predicted structures to the native structure. We quantified structural accuracy using the maximum DockQ score between a predicted structure and the corresponding native structure. DockQ measures the interface similarity between two structures ^23,24^. AlphaLink2, GRASP, and Boltz2 predicted at least one near-native structure, *i.e.*, a structure with an acceptable DockQ (DockQ > 0.23), for 36, 36, and 34 complexes, respectively (Fig. 3A, Supp Table 1) ^24^.

### Are the predicted structures stereochemically plausible?

We further assessed the stereochemical plausibility of the predicted structures using MolProbity based on the MolProbity score and the clashscore ^25^. The MolProbity score reflects the crystallographic resolution of the structure. A predicted structure is better than the average structure at that resolution if the MolProbity score is lower than the crystallographic resolution. The clashscore indicates the number of inter-atomic clashes in the predicted structure, with a clashscore less than 5 considered ideal ^25^.

The mean MolProbity scores for the predicted structures by AlphaLink2, Boltz2, and GRASP were lower than the resolution of the experimental structure for 27, 31, and 24 complexes, respectively (Fig. 3B, Supp Table 1). This indicates that for most complexes, the predicted structures are stereochemically plausible. Of the three methods, GRASP predicted structures with the lowest stereochemical plausibility. We then compared the mean clashscore of the predicted structures from each method against the clashscore of the corresponding native structure (Supp Fig. 3). For all three methods, the mean clashscore across all complexes is similar to or higher than the clashscore for the native structure, indicating that the predicted structures have similar to or more inter-atomic clashes than the native structures. Clashscores from GRASP-predicted structures were higher than those from others for several complexes in the benchmark (Fig. 3).

### Is the set of predicted structures diverse?

**Figure 4:**
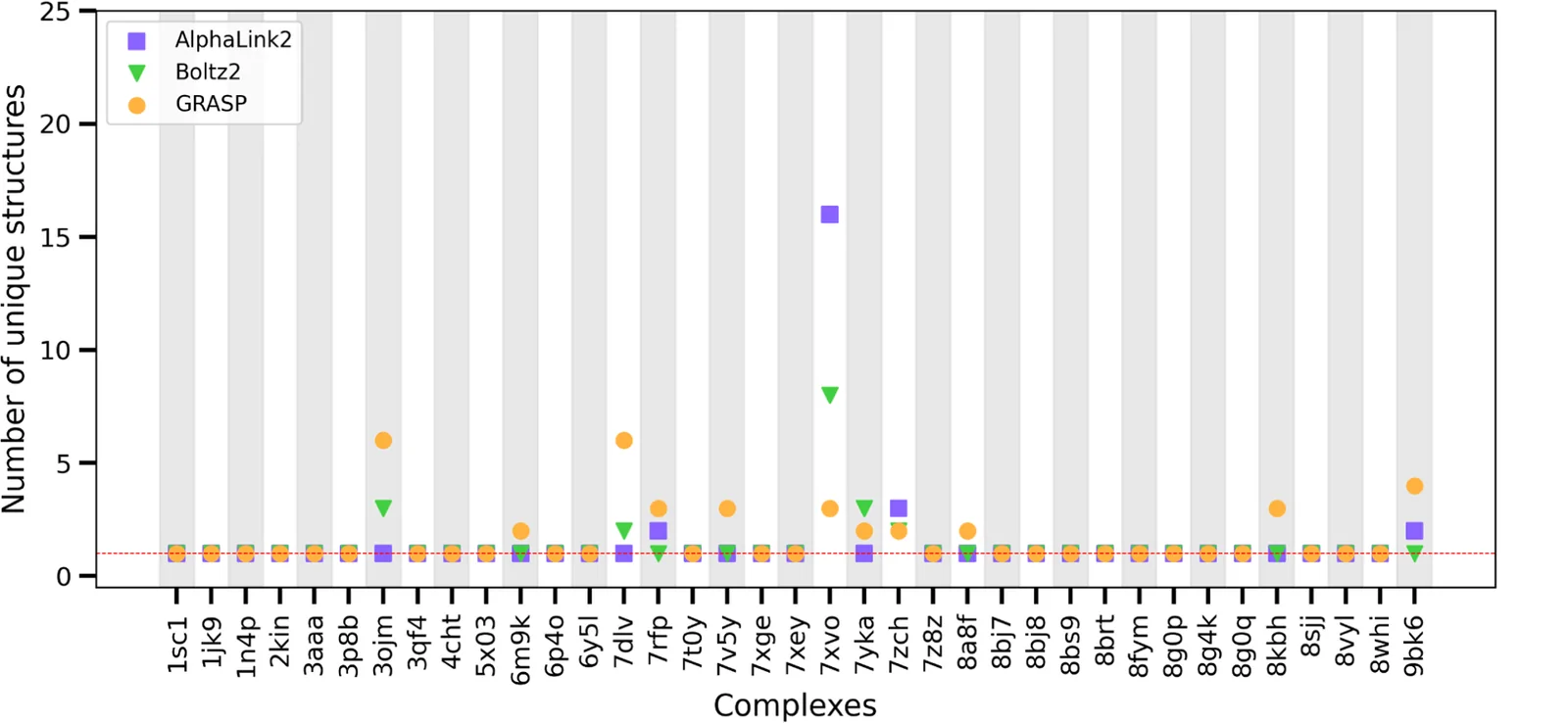
Assessing structural diversity. For each complex, the number of unique predicted structures by each method based on the DockQ score is shown for AlphaLink2 (purple, squares), Boltz2 (green, triangles), and GRASP (orange, circles).

Next, we assessed the methods based on their ability to sample a diverse set of structures, given input crosslinks. Given a sparse set of crosslinks with an upper bound of 20 Å, more than one structure may plausibly satisfy the data. To assess structural diversity, we first filtered the predicted structures based on interface similarity to obtain a set of unique structures (See Methods). We then quantified structural diversity by the number of unique structures predicted by each method for each complex. For most complexes, all three methods failed to predict a diverse set of structures (Fig. 4). GRASP predicted more than one structure for 11 complexes, whereas AlphaLink2 and Boltz2 did so for only 4 and 5 complexes, respectively, indicating that GRASP can sample a more diverse set of structures (Supp Table 1).

We further probed whether the lack of diversity results from the model being highly confident in its predictions. For this, we measured mean model confidence across the set of predicted structures by each method across all complexes. The model confidence appears to be uncorrelated with the diversity of predicted structures (Supp Fig. 4). The Boltz2 (GRASP) predicted structures always have a high (low) confidence.

However, since most crosslinks used herein are derived from a single native structure per complex, there may not exist a diverse set of configurations consistent with the crosslinks for most complexes, despite the low resolution of crosslinking data. So, we cannot completely rule out the possibility that these methods may predict alternate configurations. Therefore, we proceeded to evaluate this aspect more directly, through two other assessments described in the next sections.

### Are the AF-based IM methods robust to noise?

Next, we evaluated the robustness of the AlphaFold-based methods to noise. For this, we used a crosslinks dataset with a higher fraction of false-positive (FP) crosslinks. As noted previously, we used adversarial FP crosslinks for which the inter-residue distance exceeds 40 Å. Satisfying these FP crosslinks would require exploring configurations beyond the near-native structures; therefore, we expect the integrative modeling methods to predict a more diverse set of structures on this dataset.

**Figure 5:**
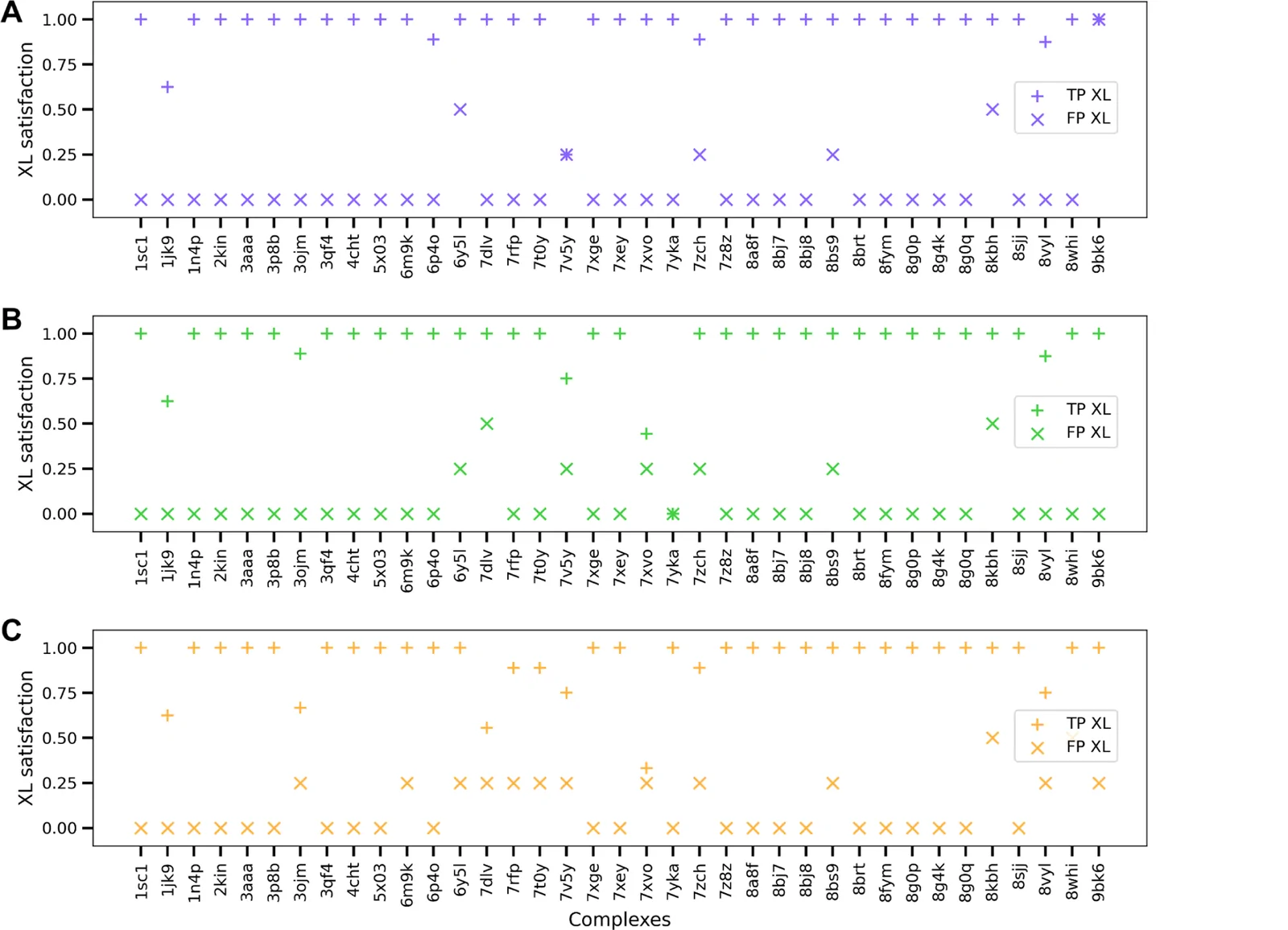
Assessing robustness to noise. For each complex, (A-C) the fraction of true positive (TP) and false positive (FP) crosslink (XL) satisfaction is shown for AlphaLink2 (purple), Boltz2 (green), and GRASP (orange).

For most complexes, all three methods satisfied the true-positive (TP) cross-links but failed to satisfy the false-positive (FP) cross-links, as measured by the maximum TP and FP cross-link satisfaction achieved by any structure (Fig. 5A,5B,5C). GRASP was slightly better at satisfying FP crosslinks, predicting structures that satisfy at least one FP crosslink for 14 complexes, whereas AlphaLink2 and Boltz2 satisfied FP crosslinks for only 6 and 7 complexes, respectively (Fig. 5A,5B,5C). GRASP also predicted a more diverse set of structures for these complexes (8/14 with more than one unique structure) than Boltz2 (4/7) and AlphaLink2 (0/6) (Supp Fig. 5A). Among the three methods, GRASP appears to be the most sensitive to crosslinks guidance. Finally, the stereochemical quality of the predicted structures does not appear to be compromised by the addition of FP crosslinks, as measured by the mean clashscore and mean Molprobity score (Supp Fig. 5B,5C).

### Does data guidance allow predicting multiple states?

The crosslink datasets used so far were derived from a single native structure per complex. As a result, it is unclear whether the limited diversity of the predicted structures reflects a limitation of the AF-based IM methods or simply the lack of alternative structures consistent with the crosslinks. We next examined the ability of these methods to predict multiple states from crosslinks. In the absence of experimental structures for multi-state protein complexes, we evaluated two monomeric proteins known to exist in apo and holo conformations ^26^.

Ribonuclease H (RNase H) is an endonuclease enzyme that degrades RNA in RNA-DNA hybrids. Calmodulin1 is part of the calcium signalling pathway and regulates various enzymes and ion channels. The TM-score between the apo and holo states for RNaseH (Calmodulin1) is 0.33 (0.43). We sought to determine whether the AF-based methods predict both the apo and holo state structures, given a combined set of crosslinks derived from both states. Additionally, we investigated whether the predictions could be guided towards a specific state using crosslinks derived exclusively from either the apo or holo state (See Methods). We assessed the performance of each method using the TM-score of the predicted structures to the apo and holo state structures.

**Figure 6:**
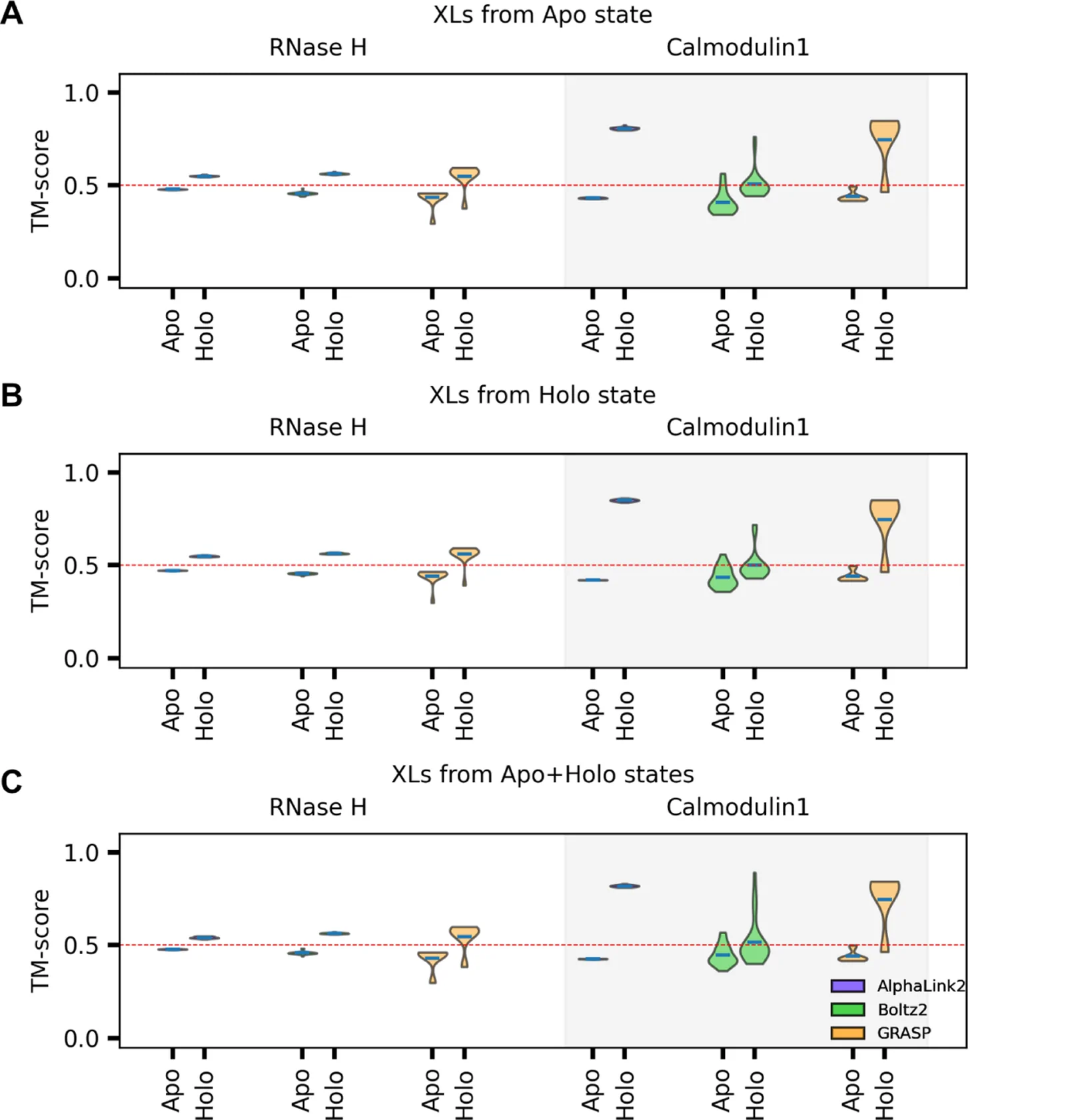
Assessing exploration of multiple states. The distribution of TM-score for the set of predicted structures with respect to the apo and holo states is shown for input crosslinks derived from (A) the apo state, (B) the holo state, and (C) the apo and holo states combined.

**Figure 7:**
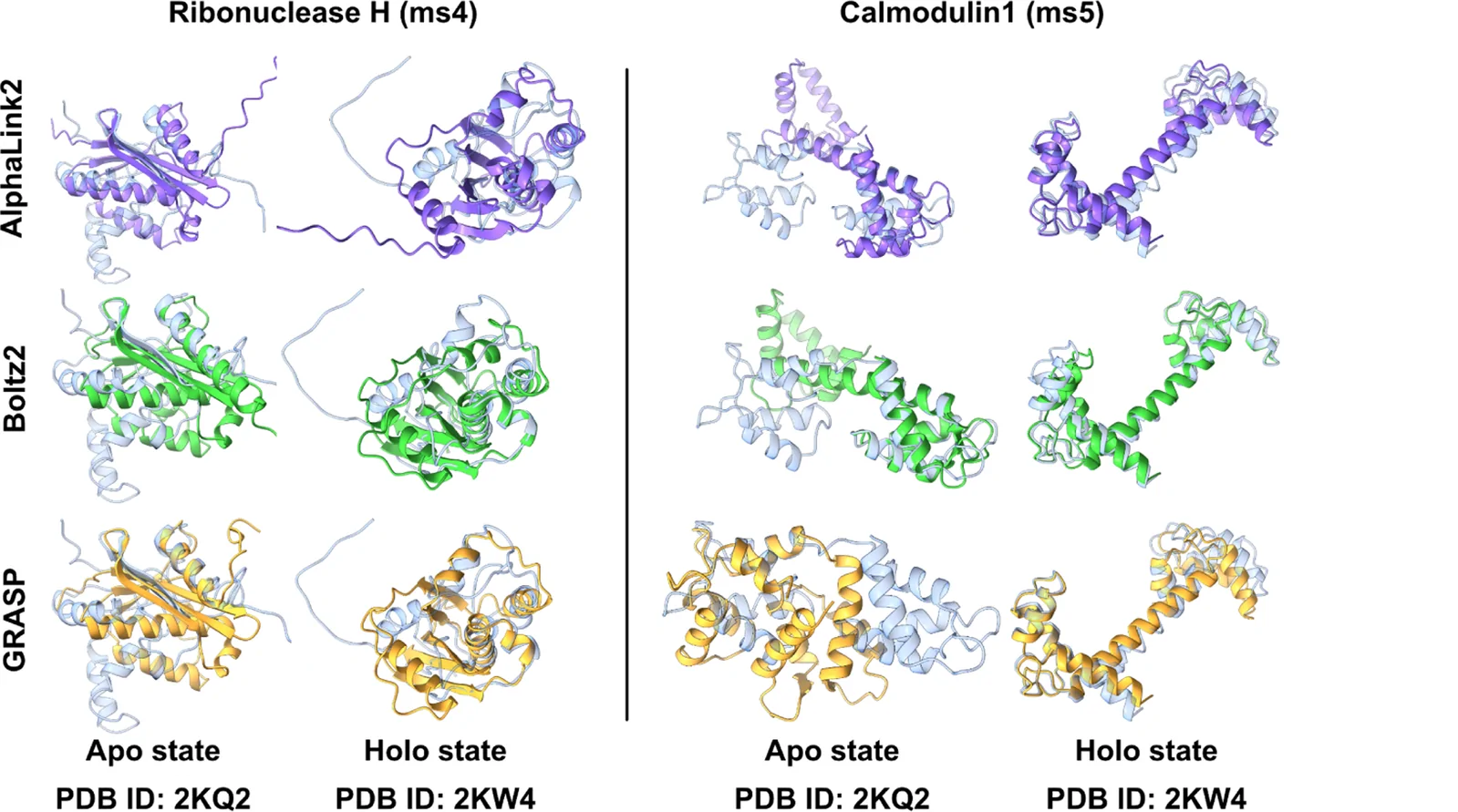
Predicted structures for multi-state proteins. For two examples of multi-state proteins, the predicted structures (solid colours) with the highest TM-scores relative to the apo and holo states (transparent) are shown for each method.

For both proteins, the predictions from all three methods were biased towards the holo state, regardless of the source of crosslinks (Fig. 6, 7, Suppl Fig. 6). AlphaLink2 and GRASP were unable to predict the apo state structure for both proteins, even when provided crosslinks derived exclusively from the apo state as input. Boltz2 predicted a few structures similar to the apo state for Calmodulin1. However, its predictions, like those of AlphaLink2 and GRASP, appear to be unaffected by the source of the input crosslinks (Fig. 6).

Besides, the distribution of the TM-score for the predicted structures is narrow, especially for AlphaLink2 (Fig. 6). Overall, the performance of these methods indicates insufficient sampling of alternative structures, plausibly due to training bias. Consequently, crosslinks guidance may be insufficient to overcome these learned biases and drive predictions towards configurations that lie outside the model’s learned structural prior.

## Discussion

The ability to incorporate restraints derived from experimental data into structure predictors like AlphaFold has opened new avenues for integrative modeling of macromolecular complexes. In this study, we evaluated the applicability for integrative modeling of three such AF-based methods – AlphaLink2, Boltz2, and GRASP – using crosslinking data. On a benchmark of 37 protein complexes, using a dataset of mostly true positive (TP) crosslinks obtained from a single experimental structure, all three methods predicted structures with high crosslink satisfaction. These structures were also accurate, as measured by the DockQ score to the native structure and the MolProbity score. Of the three methods, Boltz2 was the fastest, whereas AlphaLink2 required the most computational time (Supp Fig. 6).

However, existing AlphaFold-based methods have their limitations. First, current methods are not scalable for modeling large macromolecular assemblies, as evidenced by the limitation to obtain predictions for complexes with more than 1400 residues on a high-memory GPU ^13^. Second, the examined methods are not sensitive to crosslinks guidance. The FP crosslink satisfaction was low for most complexes when a larger number of adversarial crosslinks were introduced alongside the true-positive crosslinks. As a result of the poor sensitivity to crosslinks, these methods are unable to predict a diverse set of structures in response to adversarial crosslinks. Of the three methods, GRASP appears to be more sensitive to input crosslinks and consequently predicts a more diverse set of structures (Suppl Fig. 5B,5C). Further evidence for the limited sensitivity of the methods to the input crosslinks came from their inability to predict distinct apo or holo conformations when provided with crosslinks exclusively from the apo or holo state. This insensitivity may arise because AlphaFold’s learned structural priors may dominate the input data, potentially biasing the predictions towards dominant regions of the structural landscape. As a result, this limits exploration of alternate structures consistent with the data. However, sampling diverse structures is crucial for integrative modeling as multiple structures may fit the potentially sparse, noisy, ambiguous, and heterogeneous input data ^3^. Thus, AlphaFold-based integrative modeling methods must shift focus from simply predicting the single best structure to data-guided exploration of conformational ensembles.

## Methods

### Dataset

#### Complex benchmark

We created a benchmark dataset comprising multimeric complexes obtained from the PDB benchmark from Mirabello C. *et al.* (2024), FoldBench, PINDER-XL, AFM benchmark, and SAbDAb (Fig. 1)^27–31^. From FoldBench, we selected protein-protein, protein-peptide, and antigen-antibody complexes. From SAbDab, we selected antibody structures that were non-redundant at 60% sequence identity and had a resolution lower than 4 Å. This resulted in a total of 2804 structures of complexes. From these structures, chain termini were trimmed to remove missing residues. Further, we removed complexes larger than 1400 residues, those containing non-standard amino acids, and those containing chains with more than 1% missing residues.

#### Simulating crosslinks

Next, we simulated crosslinks using JWalk ^32^. We selected all inter-protein Lys-Lys crosslinks from JWalk with a solvent-accessible surface distance (SASD) less than 20 Å. Complexes with fewer than eight inter-protein crosslinks were removed. For complexes with more than eight inter-protein crosslinks, a random subset of eight crosslinks was selected as true positives (TP). Additionally, we added 10% adversarial false-positive (FP) crosslinks, *i.e.,* one FP crosslink (1 FP XL) per complex with a SASD of greater than 40 Å. With this, our 10% FP dataset contained nine crosslinks per complex (8 TP and 1 FP). We also created a second dataset containing a higher fraction of FP crosslinks to simulate greater noise. For each complex, we added three FP crosslinks to the existing nine crosslinks to create a dataset with 33% FP crosslinks.

#### Multi-state proteins

Due to the lack of multi-state protein complexes with fewer than 1400 residues, we use monomeric proteins known to exist in apo and holo states. We selected two such proteins from an existing multi-state protein benchmark – Ribonuclease H (PDB IDs: 2KQ2 and 2KW4) and Calmodulin1 (PDB IDs: 1DMO and 3CLN) ^26^. Of the 91 proteins in the benchmark, our selection was based on the following two criteria: (a) the TM-score between the apo and holo states must be lower than 0.5, (b) at least five mutually exclusive, state-specific crosslinks must be obtained for each of the states. We considered all 20 standard amino acids for computing crosslinks from JWalk within a maximum SASD of 20 Å. For each protein, we considered the maximum contiguous sequence in the PDB entries for the apo and holo states.

#### Predictions from the AlphaFold-based Integrative Modeling methods

For this study, we selected three AF-based methods that allow incorporating residue-level interaction data as obtained from XLMS and enable predictions for multimeric complexes: (i) AlphaLink2, (ii) Boltz2, and (iii) GRASP ^18,20,21^. To account for the variability in the predictions arising from differences in the input MSAs, we used the OpenFold MSA creation pipeline to create MSAs for all complexes in the benchmark ^33^. Complexes for which the MSA creation failed were excluded. The predictions were obtained using the default arguments for all three methods, unless stated otherwise in the paper. A total of 25 structures were predicted by each method. For three complexes (5XCT, 6IWW, 7AGF), we were unable to obtain predictions from AlphaLink2 and Boltz2, and hence these were excluded. Thus, a total of 37 complexes were considered for evaluation.

#### Data satisfaction

We compute the fraction of crosslinks satisfied by each structure in the predicted set of structures. A crosslink is considered satisfied if the Cα-Cα distance between the crosslinked residues is smaller than the maximum allowed crosslink distance of 20 Å. We account for ambiguity by considering a crosslink satisfied if any of the multiple copies of a protein in the complex satisfy the crosslink. Thus, the crosslink satisfaction for a given predicted structure, $S_{xl}$ is computed as,

$$S_{xl}=\frac{1}{M}\sum_{\left(i,j\right)}^{M}\;I\underset{a,b}{(min}\;d_{ab}(i,j)\leq d_{max}))$$

where, 𝑀 represents the set of all crosslink residue pairs $(i,j),\;d_{max}$ represents the maximum allowed crosslink distance, $d_{ab}(i,j)$ represents the distance between residue i in copy a and residue j in copy b, and I is the indicator function. For each complex, we considered the maximum crosslink satisfaction across the predicted set of structures.

#### Structural accuracy

For assessing structural accuracy, we computed the TM-score and DockQ score ^24,34^. To assess the structural accuracy of complexes, we measured the interface similarity of the predicted structures to the native structure, using the DockQ score, computed *via* the DockQ-v2 Python library ^24^. We evaluated methods based on the maximum DockQ score across their set of predicted structures for each complex. For multi-state proteins, we used the TM-score, as computed from USalign, to estimate the accuracy of each predicted structure with respect to the native apo and holo state structures ^34^.

#### Stereochemical plausibility

We used MolProbity from the Phenix suite for measuring stereochemical plausibility ^25^. We computed the MolProbity score and interatomic clashscore for all predicted structures. We evaluated the set of predicted structures from each method by their mean MolProbity score and mean clashscore.

#### Structural diversity

We measured diversity in the set of 25 predicted structures per complex per method by determining the number of structurally distinct interfaces. We computed all-vs-all pairwise DockQ scores to evaluate interface similarity between all pairs of structures, considering two structures as similar if their DockQ score exceeded 0.23 ^23,24^. We then iteratively eliminated structurally similar predictions (DockQ > 0.23) to obtain a non-redundant set of structures.

## Supplementary Material

Supplement 1

## Data Availability

The datasets created in this manuscript have been deposited to Zenodo at https://doi.org/10.5281/zenodo.22159054. The deposition contains the benchmark dataset used in the study, the predictions from Boltz2 and GRASP, and the figures presented in the paper. The scripts to create the benchmark dataset, obtain predictions from AlphaLink2, Boltz2, and GRASP, and perform analysis are available at GitHub https://github.com/isblab/af_im. Any additional information required to reanalyze the data reported in this paper is available from the lead contacts upon request.
