# Evaluation of methods for AlphaFold-based integrative modeling

**DOI:** 10.64898/2026.09.13.751323

**Published:** 2026-11-05

**Authors:** Kartik Majila, Shruthi Viswanath

## Abstract

Recent methods enable incorporation of experimental data, such as chemical crosslinks, X-ray and cryo-EM density maps, into AlphaFold to guide the predictions towards structures consistent with the input data. However, it is unclear how these methods balance the input data with AlphaFolds learned structural priors. To probe this aspect, we curated a benchmark of homomeric and heteromeric protein complexes non-redundant with the AlphaFold training set. We evaluated three methods, AlphaLink2, Boltz2, and GRASP, on the benchmark, using chemical crosslinks as input data. All methods predict structures that satisfy the crosslinks well, in the easy case, where 90% of the input crosslinks are consistent with a single structure. Yet this apparent success belies important limitations. First, for most complexes, the methods produce nearly identical predictions with and without the input crosslinks. Second, predictions remain largely unchanged from the easy case even as the fraction of false-positive crosslinks increases, even though satisfying these crosslinks would require sampling conformations that differ substantially from those predicted in the easy case. Finally, when challenged with input crosslinks from multiple states, the methods remain biased toward a single state. Together, these findings suggest that current AlphaFold-based approaches can be insufficiently sensitive to the input data, with the predictions likely driven primarily by the learned structural priors. Our study underscores the need for AF-based IM approaches that can weigh experimental evidence better and can capture structural heterogeneity. The benchmark and all code to obtain the predictions and perform the analysis are available at https://github.com/isblab/af_im.

## Full Text Availability

The license terms selected by the author(s) for this preprint version do not permit archiving in PMC. The full text is available from the preprint server.

