# Supplementary material for: Evaluation of methods for AlphaFold-based integrative modeling
Source: bioRxiv. 2026 Sep 16:2026.09.13.751323. Preprint. [Version 1] doi: 10.64898/2026.09.13.751323 (PMC13596301; doi:10.64898/2026.09.13.751323)
Supplement: Supplementary file 1 [file media-1.pdf]

Supplementary material

Evaluation of methods for AlphaFold-based integrative modeling

Kartik Majila[1,*] and Shruthi Viswanath[1,*,+]

[1]National Centre for Biological Sciences, Tata Institute of Fundamental Research, Bangalore, Karnataka, India 560065.

[*]Corresponding authors.
Email: kartikm@ncbs.res.in (KM); shruthiv@ncbs.res.in (SV)

[+]Lead contact
Shruthi Viswanath. Email: shruthiv@ncbs.res.in

Keywords: AlphaFold, Integrative modeling, Macromolecular assemblies, Structure prediction

Supplementary figures

Supp Figure 1: Maximum crosslink satisfaction for guided and unguided predictions. For Boltz2 (green, triangle) and GRASP (orange, circle), we show the maximum crosslink satisfaction achieved by a structure in the set of predicted structures for each complex, comparing guided vs unguided predictions.

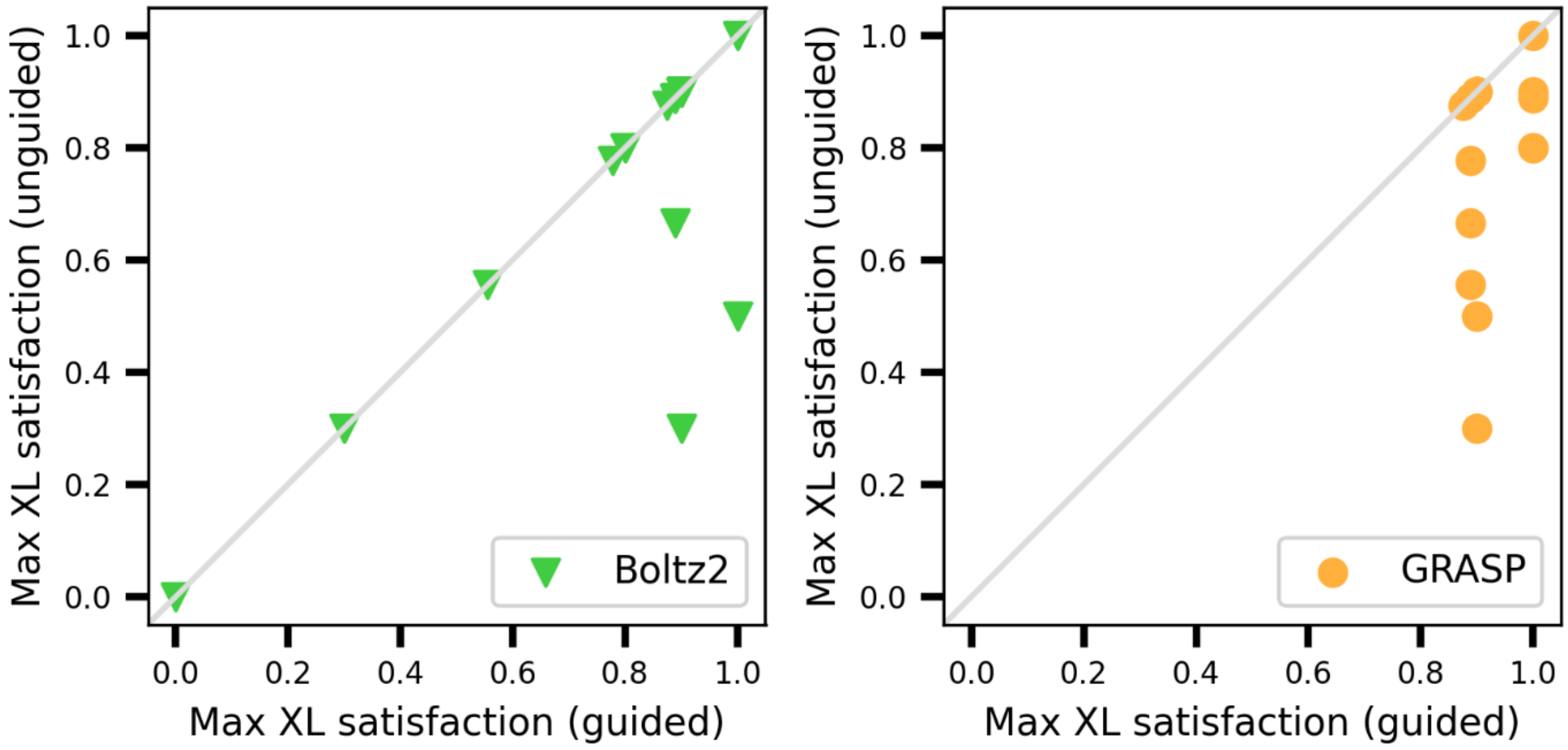

Supp Figure 2: Distribution of crosslink satisfaction for guided and unguided predictions. The distribution of crosslink satisfaction in the predicted set for each complex, comparing restraint-guided vs unguided predictions for (A) Boltz2 and (B) GRASP.

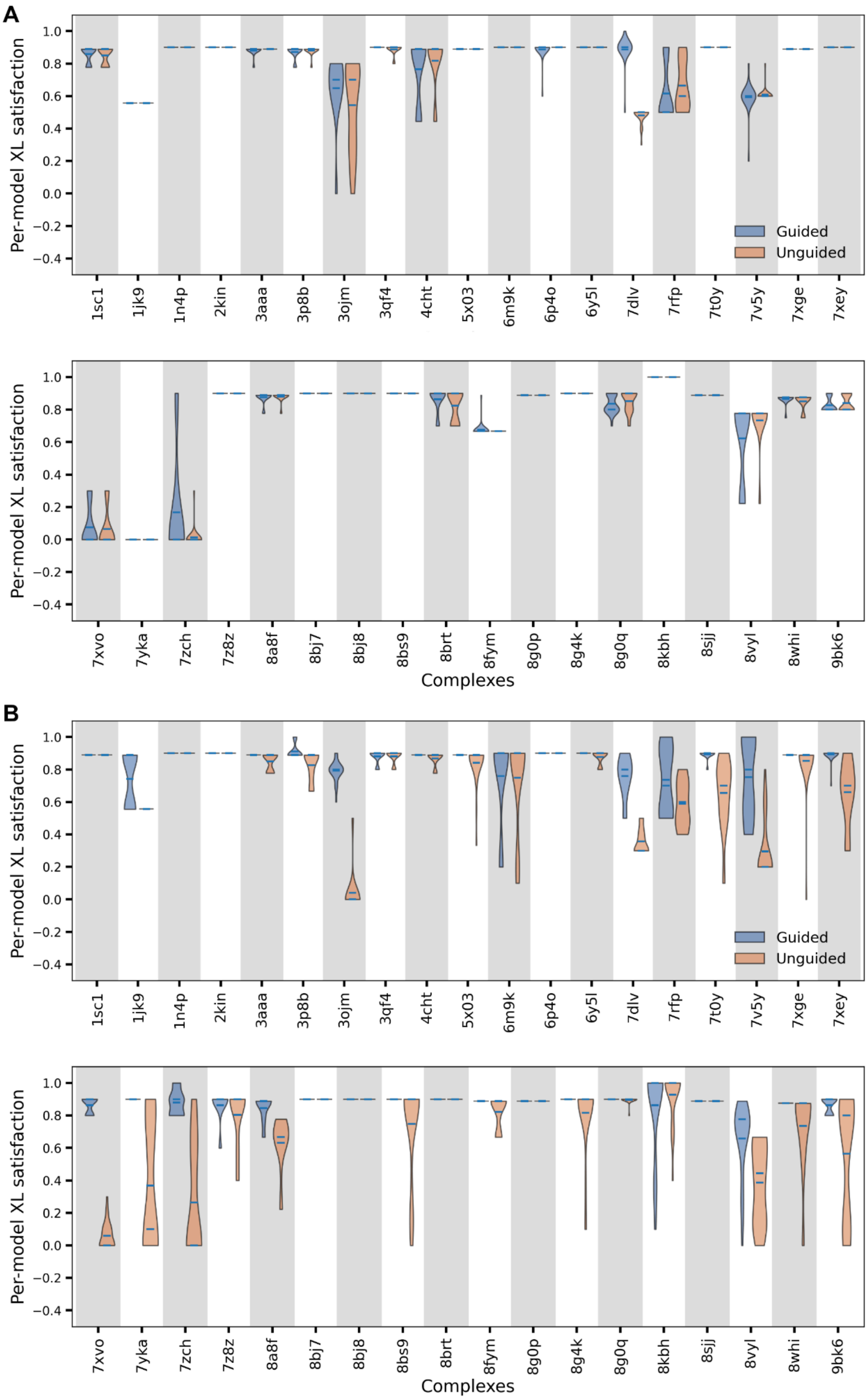

Supp Figure 3: Assessing mean clashscore. The mean clashscore of the predicted set of structures vs the clashscore for the native structure for each complex for AlphaLink2 (purple, square), Boltz2 (green, triangle), and GRASP (orange, circle) is shown.

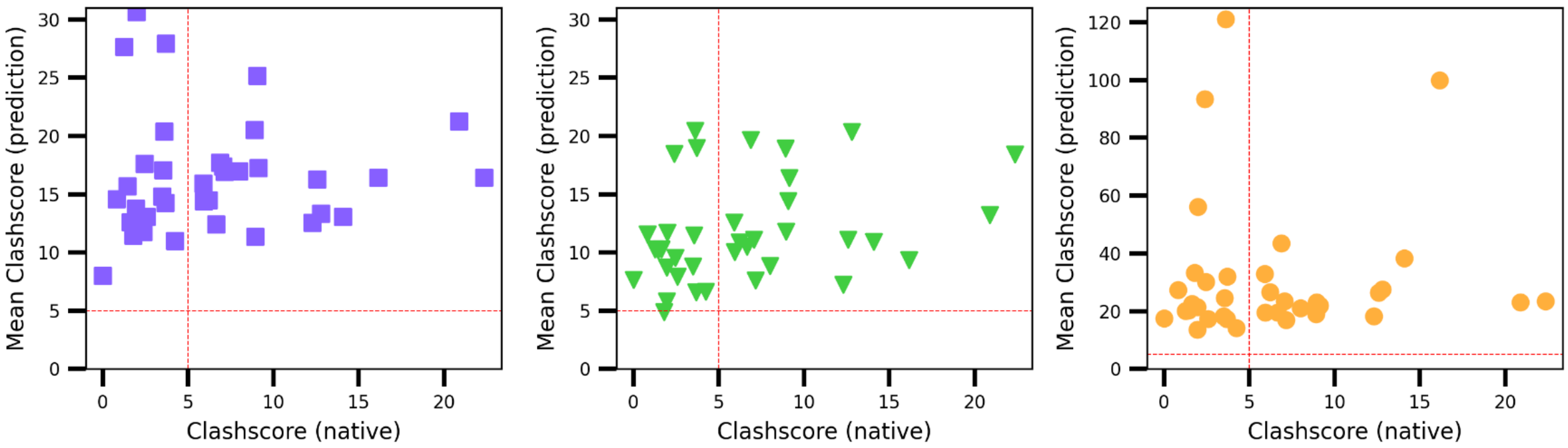

Supp Figure 4: Assessing predicted model confidence. The mean model confidence across the predicted set of structures by AlphaLink2 (purple, square), Boltz2 (green, triangle), and GRASP (orange, circle) for each complex is shown.

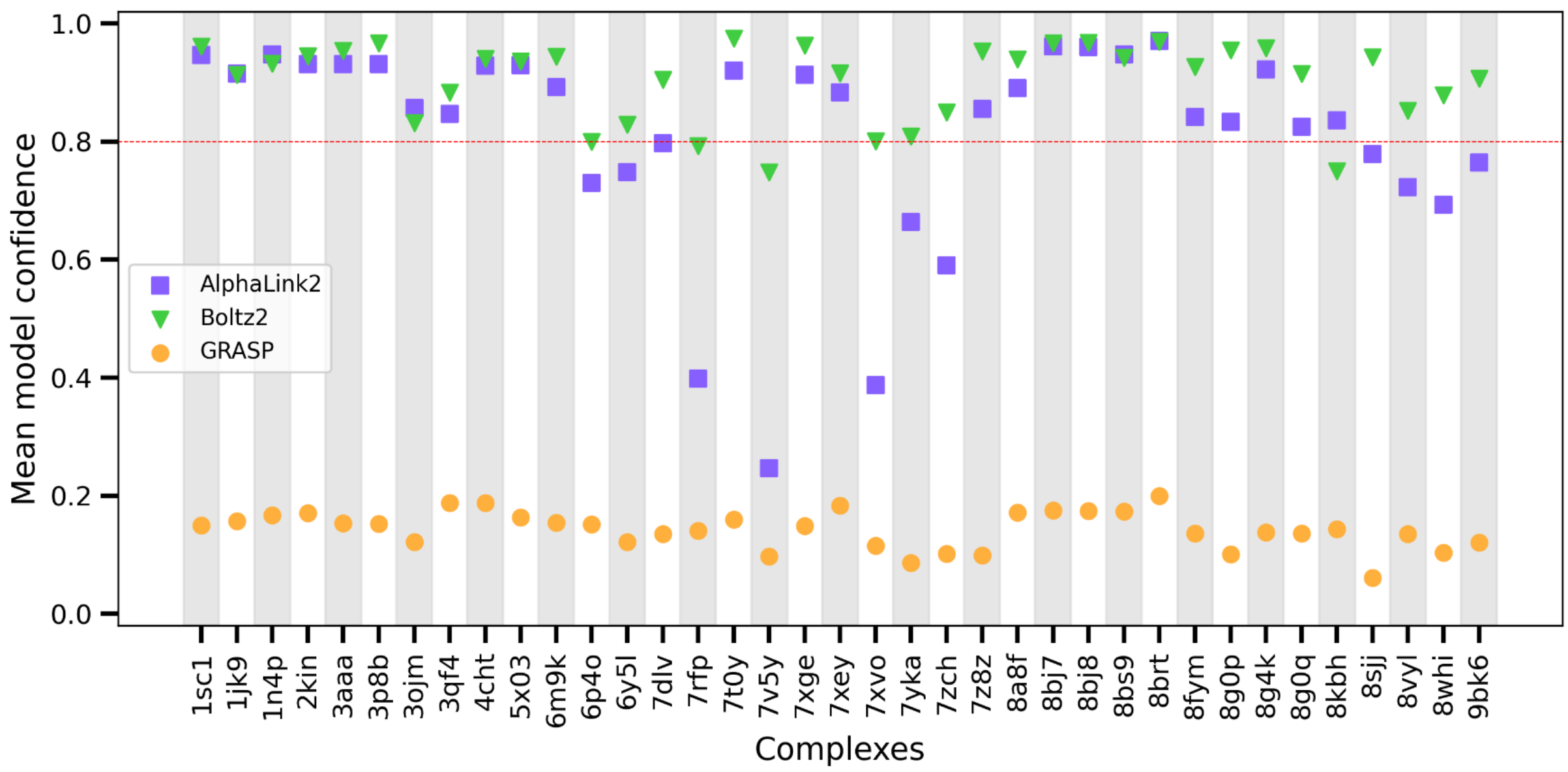

Supp Figure 5: Robustness to false positive crosslinks. For the predictions obtained from AlphaLink2 (purple, square), Boltz2 (green, triangle), and GRASP (orange, circle) using 10% vs 33% false positive (FP) crosslinks, (A) the number of unique predicted structures based on DockQ and (B) the mean molProbity score are shown.

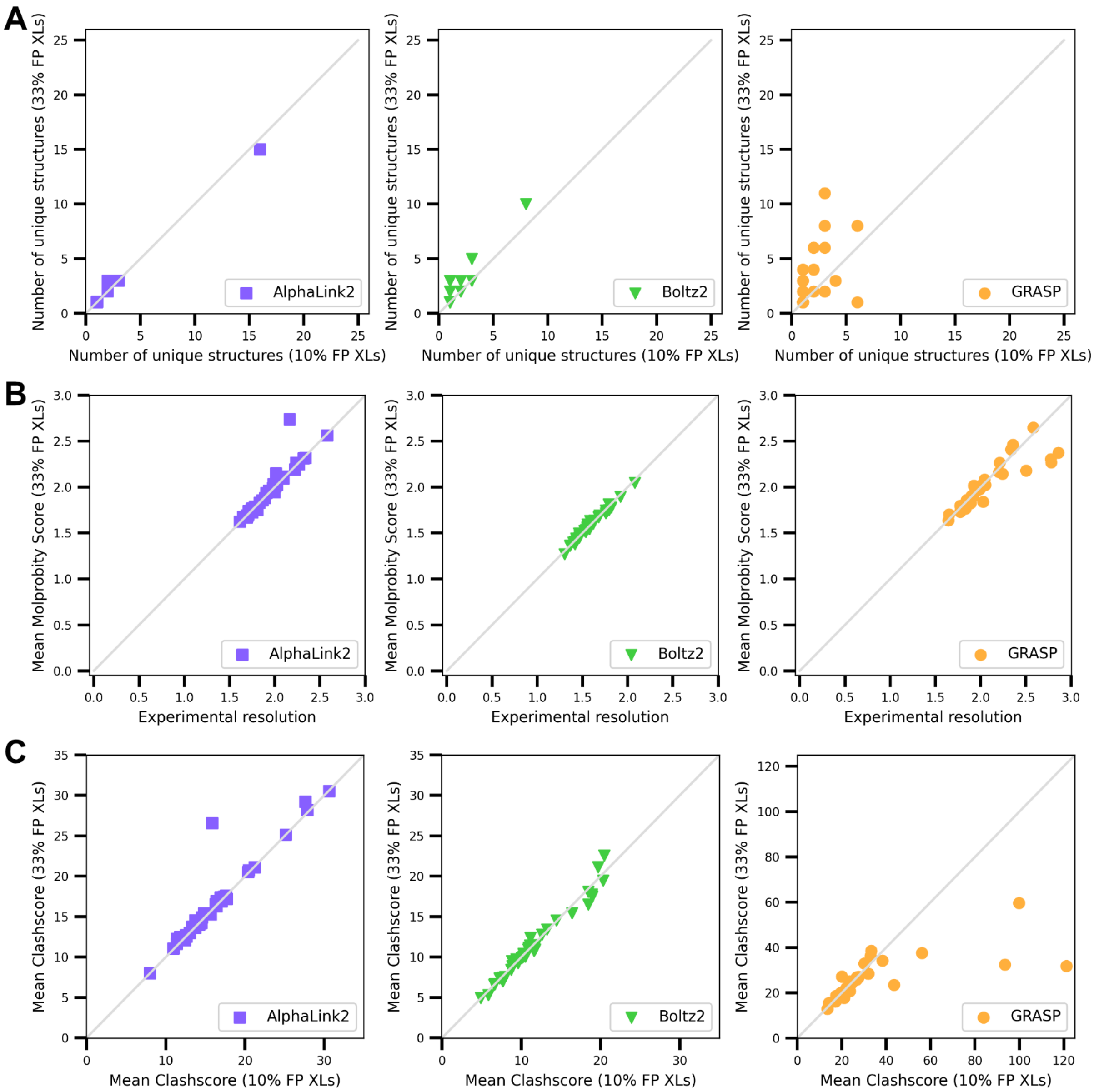

Supp Figure 6: Time taken for predictions. We measure the mean time taken (in hours) by each method across the benchmark. AlphaLink2 (purple), Boltz2 (green), and GRASP (orange). Runtimes were computed on an AMD EPYC 7763 64-Core Processor, with 1024 GB RAM, and an A6000 GPU.

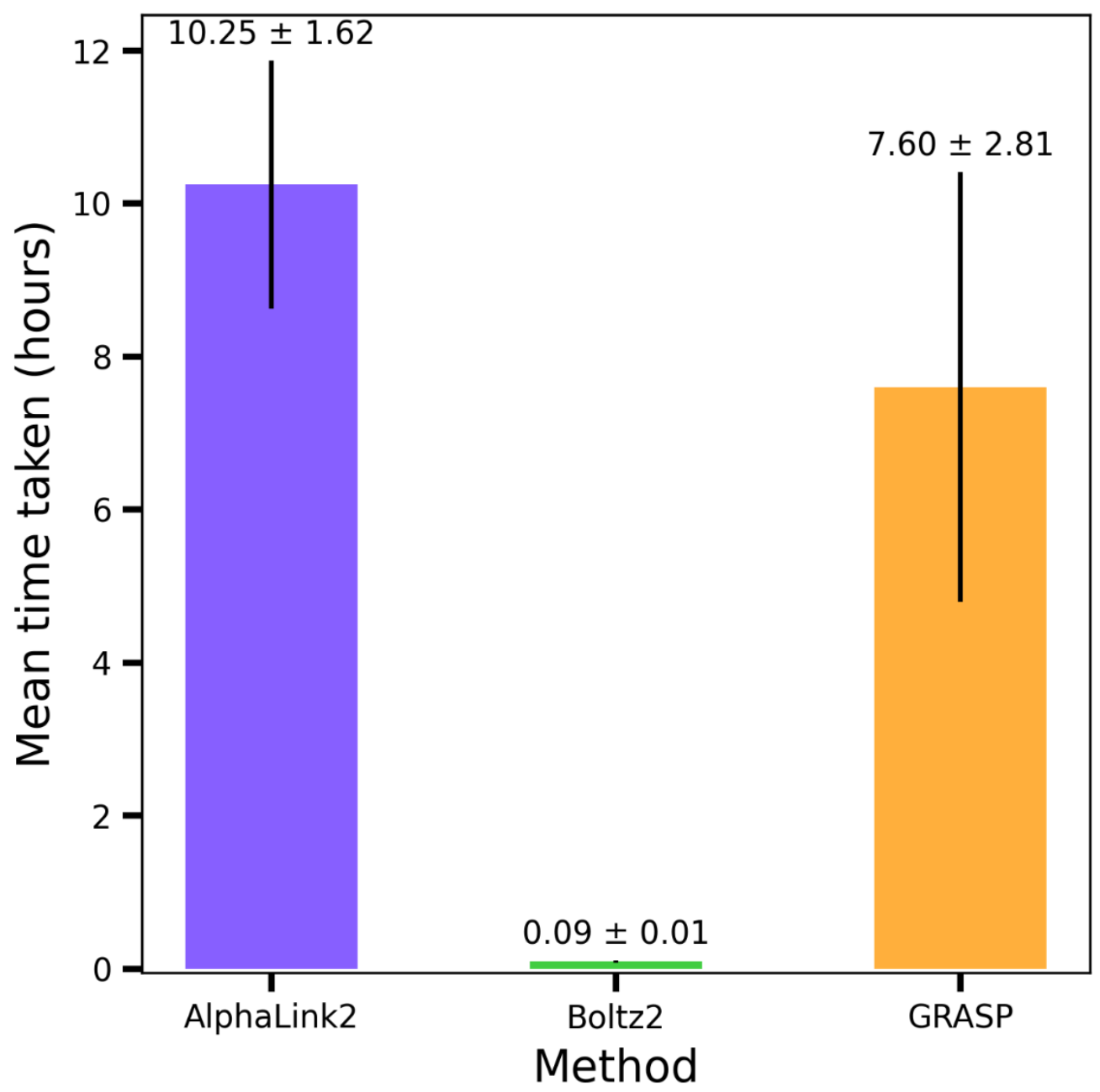

Supplementary tables

Supp Table 1: Assessing the performance of AlphaFold-based integrative modeling methods. For each complex in the benchmark, the crosslink satisfaction, DockQ, number of unique structures, Molprobity score, and clashscore are reported for AlphaLink2, Boltz2, and GRASP.

Complex	Method	Crosslink satisfaction		DockQ		Number of structures with unique interface	Mean Molprobity score	Mean clashscore
		Max	Mean	Max	Mean			
**1sc1**	Alphalink2	0.889	0.889	0.6	0.6	1	1.864	14.396
	Boltz2	0.889	0.858	0.5	0.5	1	1.618	10.032
	Grasp	0.889	0.889	0.6	0.6	1	1.99	19.636
**1jk9**	Alphalink2	0.556	0.556	0.5	0.404	1	1.78	16.398
	Boltz2	0.556	0.556	0.5	0.452	1	1.758	18.446
	Grasp	0.889	0.742	0.5	0.44	1	2.028	23.556
**1n4p**	Alphalink2	0.9	0.9	0.9	0.9	1	1.893	17.226
	Boltz2	0.9	0.9	0.8	0.62	1	1.919	16.393
	Grasp	0.9	0.9	0.9	0.836	1	1.956	22.031
**2kin**	Alphalink2	0.9	0.9	0.8	0.748	1	1.694	12.532
	Boltz2	0.9	0.9	0.8	0.728	1	1.397	7.267
	Grasp	0.9	0.9	0.8	0.708	1	1.883	18.3
**3aaa**	Alphalink2	0.889	0.787	0.7	0.7	1	1.711	14.23
	Boltz2	0.889	0.88	0.7	0.7	1	1.361	6.585

	Grasp	0.889	0.889	0.8	0.8	1	1.772	17.386
**3p8b**	Alphalink2	0.889	0.889	0.8	0.708	1	2.008	16.965
	Boltz2	0.889	0.871	0.9	0.752	1	1.47	8.839
	Grasp	1	0.911	0.9	0.664	1	1.833	21.093
**3ojm**	Alphalink2	0.9	0.804	0.7	0.544	1	2.072	13.335
	Boltz2	0.8	0.648	0.3	0.152	3	1.817	20.35
	Grasp	0.9	0.792	0.7	0.268	6	2.211	27.55
**3qf4**	Alphalink2	0.9	0.9	0.8	0.8	1	2.583	21.22
	Boltz2	0.9	0.9	0.4	0.4	1	1.629	13.238
	Grasp	0.9	0.88	0.7	0.56	1	2.012	23.052
**4cht**	Alphalink2	0.889	0.889	0.8	0.796	1	1.909	13.041
	Boltz2	0.889	0.764	0.8	0.744	1	1.432	7.946
	Grasp	0.889	0.889	0.8	0.712	1	1.845	17.428
**5x03**	Alphalink2	0.889	0.889	0.7	0.7	1	1.751	11.365
	Boltz2	0.889	0.889	0.7	0.688	1	1.586	11.783
	Grasp	0.889	0.889	0.7	0.684	1	1.847	23.058
**6m9k**	Alphalink2	0.9	0.9	0.8	0.76	1	1.697	15.667
	Boltz2	0.9	0.9	0.8	0.8	1	1.532	10.206
	Grasp	0.9	0.76	0.8	0.616	2	1.796	20.232
**6p4o**	Alphalink2	0.8	0.68	0.2	0.2	1	2.319	20.489
	Boltz2	0.9	0.888	0.2	0.136	1	1.793	18.926
	Grasp	0.9	0.9	0.3	0.3	1	1.986	19.01
**6y5l**	Alphalink2	0.9	0.828	0.4	0.272	1	2.165	15.884

	Boltz2	0.9	0.9	0.4	0.296	1	1.583	12.601
	Grasp	0.9	0.9	0.1	0.076	1	2.338	32.886
**7dlv**	Alphalink2	0.9	0.868	0.8	0.684	1	1.782	17.695
	Boltz2	1	0.888	0.7	0.648	2	1.789	19.67
	Grasp	0.9	0.76	0.6	0.328	6	2.503	43.497
**7rfp**	Alphalink2	0.8	0.58	0.3	0.128	2	2.267	20.384
	Boltz2	0.9	0.616	0.4	0.284	1	2.08	20.478
	Grasp	1	0.736	0.3	0.132	3	2.781	121.186
**7t0y**	Alphalink2	0.9	0.9	0.9	0.78	1	2.095	11.449
	Boltz2	0.9	0.9	0.9	0.872	1	1.429	4.883
	Grasp	0.9	0.892	0.3	0.188	1	2.58	33.296
**7v5y**	Alphalink2	0.2	0.2	0.8	0.72	1	1.755	11.778
	Boltz2	0.8	0.592	0.3	0.196	1	1.756	18.458
	Grasp	1	0.752	0.3	0.124	3	2.86	93.426
**7xge**	Alphalink2	0.889	0.889	0.9	0.84	1	2.238	16.9
	Boltz2	0.889	0.889	0.9	0.9	1	1.413	7.634
	Grasp	0.889	0.889	0.9	0.88	1	1.774	17.034
**7xey**	Alphalink2	0.9	0.848	0.7	0.588	1	2.224	17.394
	Boltz2	0.9	0.9	0.8	0.8	1	1.56	11.108
	Grasp	0.9	0.892	0.6	0.56	1	2.052	23.438
**7xvo**	Alphalink2	0.9	0.36	0.7	0.184	16	1.81	12.414
	Boltz2	0.3	0.076	0	0	8	1.536	10.496
	Grasp	0.9	0.864	0.7	0.408	3	1.94	19.55

**7yka**	Alphalink2	0.9	0.9	0.3	0.3	1	1.936	27.625
	Boltz2	0	0	0	0	3	1.521	10.266
	Grasp	0.9	0.9	0.8	0.264	2	1.924	20.075
**7zch**	Alphalink2	0.8	0.728	0.4	0.26	3	2.017	16.43
	Boltz2	0.9	0.168	0.5	0.072	2	1.473	9.356
	Grasp	1	0.88	0.4	0.184	2	2.776	99.886
**7z8z**	Alphalink2	0.9	0.9	0.8	0.72	1	2.027	27.904
	Boltz2	0.9	0.9	0.8	0.716	1	1.771	18.976
	Grasp	0.9	0.864	0.8	0.704	1	2.001	32.012
**8a8f**	Alphalink2	0.889	0.889	0.6	0.6	1	2.1	17.624
	Boltz2	0.889	0.876	0.6	0.556	1	1.679	9.568
	Grasp	0.889	0.844	0.6	0.444	2	2.356	30.17
**8bj7**	Alphalink2	0.9	0.9	0.9	0.9	1	1.762	14.481
	Boltz2	0.9	0.9	0.9	0.9	1	1.553	10.865
	Grasp	0.9	0.9	0.9	0.796	1	1.898	26.693
**8bj8**	Alphalink2	0.9	0.9	0.9	0.9	1	1.755	14.58
	Boltz2	0.9	0.9	0.9	0.9	1	1.578	11.591
	Grasp	0.9	0.9	0.9	0.8	1	1.914	27.328
**8bs9**	Alphalink2	0.9	0.9	0.9	0.9	1	1.689	13.715
	Boltz2	0.9	0.9	0.9	0.812	1	1.465	8.71
	Grasp	0.9	0.9	0.8	0.716	1	1.888	21.472
**8brt**	Alphalink2	0.9	0.9	0.9	0.9	1	1.744	12.608
	Boltz2	0.9	0.864	0.9	0.9	1	1.541	10.288

	Grasp	0.9	0.9	0.9	0.88	1	1.989	22.54
**8fym**	Alphalink2	0.889	0.889	0.4	0.324	1	1.834	17.05
	Boltz2	0.889	0.676	0.3	0.232	1	1.572	11.497
	Grasp	0.889	0.889	0.6	0.392	1	2.004	24.519
**8g0p**	Alphalink2	0.889	0.889	0.6	0.6	1	1.617	10.982
	Boltz2	0.889	0.889	0.9	0.88	1	1.363	6.668
	Grasp	0.889	0.889	0.8	0.684	1	1.65	14.156
**8g4k**	Alphalink2	0.9	0.9	0.8	0.724	1	1.667	7.989
	Boltz2	0.9	0.9	0.8	0.7	1	1.416	7.644
	Grasp	0.9	0.9	0.8	0.692	1	1.808	17.478
**8g0q**	Alphalink2	0.9	0.9	0.6	0.58	1	2.342	25.167
	Boltz2	0.9	0.836	0.6	0.556	1	1.685	14.437
	Grasp	0.9	0.9	0.6	0.544	1	2.201	21.624
**8kbh**	Alphalink2	1	1	0.8	0.8	1	1.712	16.283
	Boltz2	1	1	0.8	0.752	1	1.556	11.121
	Grasp	1	0.864	0.8	0.668	3	1.946	26.52
**8sjj**	Alphalink2	0.889	0.889	0.7	0.7	1	1.65	13.733
	Boltz2	0.889	0.889	0.8	0.7	1	1.302	5.83
	Grasp	0.889	0.889	0.8	0.688	1	1.641	13.586
**8vyl**	Alphalink2	0.778	0.751	0.7	0.528	1	1.978	13.034
	Boltz2	0.778	0.622	0.5	0.364	1	1.552	10.916
	Grasp	0.889	0.658	0.5	0.348	1	2.044	38.262
**8whi**	Alphalink2	0.875	0.875	0.5	0.496	1	1.999	14.8

	Boltz2	0.875	0.865	0.5	0.328	1	1.461	8.794
	Grasp	0.875	0.875	0.5	0.436	1	1.852	18.33
**9bk6**	Alphalink2	0.9	0.868	0.8	0.768	2	1.986	30.649
	Boltz2	0.9	0.828	0.7	0.688	1	1.572	11.71
	Grasp	0.9	0.864	0.7	0.576	4	2.243	56.174
